# Development and validation of retrospective electronic frailty index using operational data of aged care homes

**DOI:** 10.1186/s12877-022-03616-0

**Published:** 2022-12-01

**Authors:** Tabinda Sarwar, Antonio Jose Jimeno Yepes, Xiuzhen Zhang, Jeffrey Chan, Irene Hudson, Sarah Evans, Lawrence Cavedon

**Affiliations:** 1 School of Computing Technologies, RMIT University, Melbourne, Australia; 2 Mathematical Sciences, School of Science, RMIT University, Melbourne, Australia; 3 Telstra Health, Melbourne, Australia

**Keywords:** Frail elderly, Frailty index, Mortality, Health deterioration, Aged care homes, Electronic health records

## Abstract

**Background:**

Although elderly population is generally frail, it is important to closely monitor their health deterioration to improve the care and support in residential aged care homes (RACs). Currently, the best identification approach is through time-consuming regular geriatric assessments. This study aimed to develop and validate a retrospective electronic frailty index (reFI) to track the health status of people staying at RACs using the daily routine operational data records.

**Methods:**

We have access to patient records from the Royal Freemasons Benevolent Institution RACs (Australia) over the age of 65, spanning 2010 to 2021. The reFI was developed using the cumulative deficit frailty model whose value was calculated as the ratio of number of present frailty deficits to the total possible frailty indicators (32). Frailty categories were defined using population quartiles. 1, 3 and 5-year mortality were used for validation. Survival analysis was performed using Kaplan-Meier estimate. Hazard ratios (HRs) were estimated using Cox regression analyses and the association was assessed using receiver operating characteristic (ROC) curves.

**Results:**

Two thousand five hundred eighty-eight residents were assessed, with an average length of stay of 1.2 ± 2.2 years. The RAC cohort was generally frail with an average reFI of 0.21 ± 0.11. According to the Kaplan-Meier estimate, survival varied significantly across different frailty categories (*p* < 0.01). The estimated hazard ratios (HRs) were 1.12 (95% CI 1.09–1.15), 1.11 (95% CI 1.07–1.14), and 1.1 (95% CI 1.04–1.17) at 1, 3 and 5 years. The ROC analysis of the reFI for mortality outcome showed an area under the curve (AUC) of ≥0.60 for 1, 3 and 5-year mortality.

**Conclusion:**

A novel reFI was developed using the routine data recorded at RACs. reFI can identify changes in the frailty index over time for elderly people, that could potentially help in creating personalised care plans for addressing their health deterioration.

**Supplementary Information:**

The online version contains supplementary material available at 10.1186/s12877-022-03616-0.

## Background

Aging generally introduces risk of developing health problems and related risks. Frailty is defined as a multidimensional geriatric syndrome characterised by a decrease in physiological reserve, which tends to increase with advancing age [1]. Frail old people often experience poor health conditions, e.g., chronic pain, hearing impairments, poor cognition, etc., and generally are at risk of disability, falls, hospitalisation, admission to nursing or high care residential aged care homes (RACs), and mortality. Hence, it is highly important to identify these poor health conditions to develop care and intervention plans to minimize the health risks.

In the clinical/hospital domain, many frailty indices (FIs) have been developed to quantify the accumulation of medical, functional, and psychosocial deficits [1–7]. FI ranges from 0 to 1, which is calculated as the proportion of accumulated deficits to the total number of health variables (potential deficits). The FIs found in the literature mainly differ in the number of health domains and deficits. Frailty in elderly individuals is generally identified using comprehensive geriatric assessments (CGA), which is a multidisciplinary diagnostic process to evaluate medical, functional, psychological and social capabilities [8]. The information gained from a CGA allows creation of the intervention plan or modification of the care plan to manage or contain any health deficits. The positive outcomes of CGA in primary care include slowing down functional decline and reduction of hospitalisations [9]. These assessments could be a time-consuming and challenging process requiring specialised equipment in a clinical setting [8]. Home-based CGA is an alternative to clinical CGA for the elderly in RACs for their care management to improve their quality of life [10]. There are many barriers and challenges to the implementation of CGAs in community settings [11], but in the context of early identification of health risks, it is difficult to identify the time for CGA to meet the needs of individuals as the health status could change rapidly and unexpectedly in the elderly [12]. Regardless of the challenges and limitations, CGA is a gold standard for the management of frailty [8, 13].

To quantify frailty of the elderly in RACS and identify any early changes in their health status, retrospective electronic FIs (reFIs) have been developed that utilise the electronic health records (EHRs) routinely collected at hospitals [14–18]. These electronic FIs (eFIs) are referred to as “retrospective” as they rely on historical health data of the patients, instead of relying on CGAs. These reFIs have successfully shown that moderate to severe frail individuals are at risk of adverse events such as hospitalisations, RAC admission, and mortality. In the RAC setting, computing frailty should be given much importance considering that an adverse event, i.e., admission to RAC, has already taken place and the individuals now require greater care and attention. We focus on retrospective electronic FIs since measuring frailty in the RAC setting still relies on CGAs and interviews [19–24], which implies that frailty cannot be regularly recorded at short intervals for residents to monitor their health conditions.

The Australian government has implemented an aged care accreditation system called the Australian Aged Care Quality and Safety Commission (AACQSC), which determines and assesses the quality of services provided by RACs. RACs regularly record daily routine data of their residents to meet the quality standards^1^, ^2^, ^3^. These records capture multiple aspects of their daily life and health/medical condition, including medication, food intake, mobility, assistance required with daily activities, sleep, weight, comorbidities, etc^3^, 4. This data is primarily used for AACQSC quality assessment^5^, but this rich data has the potential for early detection of health anomalies and in turn, early intervention to address the underlying health problems. Studies have also demonstrated that interventions can reverse or delay the progression of the frailty [25, 26]. It is worth noting that other countries such as United Kingdom, Germany, France, etc. also share similar principles to Australia to regulate the quality of living at RACs [27, 28].

Here, we propose a reFI using EHRs recorded in RAC settings that could be used to quantify regularly monitor the health status of the residents and identify early signs of health deterioration. In this paper, we will demonstrate that daily routine operational data recorded in RACs could be mapped to the health deficits for computing reFI, where that data is in the form of progress notes, observation charts, and assessments (recorded as per individual’s need). Recent studies [21, 29, 30] have evaluated frailty using similar data acquired from aged care homes but these studies also relied on performing assessments, interviews, and filling questionnaires to study frailty. Ambagtsheer et al. [30] used the Aged Care Funding Instrument (ACFI) assessment, which may not be regularly recorded for every resident. The primary purpose of this assessment is to request funds from the Australian government, but the ACFI assessment will be replaced by a new funding instrument in the near future^6^. Hence, this assessment will not be available to compute frailty in the future. In our study, the computation of the reFI is independent of the funding instruments and relies only on the daily routine operational data that is collected by RACs to comply with AACQSC quality standards. The proposed reFI computation could therefore be applied to other RACs that collect daily routine data, including RACs outside of Australia. We would also empathise that the proposed data-driven reFI is not a replacement for CGAs but is a complementary tool that could be used to automate the process of quantifying and monitoring the frailty among elderly living at RACs.

## Methods

### Study design

We conducted a retrospective, cross-sectional study using anonymised EHRs from aged care homes, operated by the Royal Freemasons Benevolent Institution (RFBI), based in New South Wales, Australia. The dataset corresponded to 26 RACs that currently have a total capacity of 1160 beds. The Human Research Ethics Committee of RMIT University (Project 23,257), Australia provided Ethics approval for the study.

### Data description

To comply with the quality standards of AACQSC, RACs record the daily routine data of residents. RFBI records this data under three major categories: progress notes, observation charts, and assessment forms. Progress notes represent the everyday progress or state of the resident in the form of free text, potentially supplemented by observation charts or assessments. An observation chart represents a short electronic document that records the pre-defined parameters associated with the state of the resident. Unlike the clinical domain, this is not restricted to vital signs or other physiological parameters. It also includes information such as bowel movements, pain, activities of daily life, and wound status. Assessments fall under the category of CGA, which is used to assess the risk of deterioration or state of the resident after an adverse event has occurred. Observation charts are frequently recorded, whereas assessments are recorded based on the individual resident’s needs. It should be noted that these assessments can be conducted at any point of a resident’s stay. Further details of the data recorded under these categories are reported in Table 1.

**Table 1:** Data recorded by RFBI aged care home

Category	Examples
Progress notes	There are multiple types of progress notes, but 3 most commonly used at RFBI are: 1. User-entered notes recorded by nurses or caretakers 2. Automated notes generated after charts or assessments 3. Medication note when PRN (pro re nata meaning “as needed”) is administered
Observation Charts	Most frequently recorded observation charts include: 1. Medication 2. Bowel Chart 3. ADL Chart 4. Blood Glucose Chart 5. Blood Pressure Chart 6. Weight Chart 7. Food Intake Chart 8. Fluid Intake Chart 9. Vital Signs Chart 10. Fluid Output Chart 11. Urinary Chart 12. Pain Chart 13. Behaviour Chart 14. Wound Chart 15. Neurological Observation Chart
Assessments	Most frequently recorded assessments include: 1. Accident/Incident Report 2. Behaviour Assessment 3. Blood Glucose Monitoring Chart 4. Physical Assessment 5. Mobility Assessment 6. Medical History 7. Medication Incident Report 8. Infection Report 9. Meals, Drinks and Nutrition Assessment 10. Wound Assessment 11. Physio-OT (Occupational Therapy) Assessment 12. Daily Fluid Balance Chart 13. Skin Integrity Assessment 14. Wound Dressing Chart 15. Falls Risk Assessment Tool (FRAT) 16. Pain Assessment 17. Personal Hygiene Assessment

### Retrospective electronic frailty index

The reFI was based on the cumulative deficit model of frailty [3, 31], which was calculated as the proportion of accumulated deficits to the total number of frailty deficits. The frailty deficits considered were adapted from 36 deficits identified by Clegg et al. using primary care data [14] following the published well-established frailty framework [1, 4, 31, 32] where deficit satisfied the three basic criteria of inclusion: is biologically and physiologically sensible, accumulates with age, and does not saturate too early. To ensure that the adapted deficits were comprehensive and comparable with the clinical reFI, screening sessions were conducted with experts in geriatric medicine, nursing and aged care who reviewed the reFI deficits. In the final reFI, eleven domains were identified that consisted of 32 frailty deficits or syndromes (Table 2, further details are reported in Additional file 1). It should be noted that “*housebound*” and “*requirement for care*” deficits from Clegg’s list applied to all the residents at RACs i.e., residents in RACs are generally housebound and may require assistance for their daily routine work [33, 34]. So “*requirement for care*” in terms of assistance was reflected in the selected frailty deficits for RACs, whose details are found in Additional file 1.

**Table 2:** List of frailty deficits contained in reFI for residential aged care homes along with their data sources in the RFBI RAC setting

Ind.	Frailty Domain	Deficit No.	Frailty Deficit	Data Source
**1**	**Chronic and Acute Diseases**	1	Respiratory	Medical History, Progress Notes
		2	Cardiac	
		3	Neurological	
		4	Renal	
		5	Cancer	
		6	Peripheral vascular disease	
		7	Thyroid	
**2**	**Blood-specific Diseases**	8	Diabetes	Medical History, Progress Notes
		9	Hypertension or Hypotension	
**3**	**Bone-specific Diseases**	10	Osteoporosis	
		11	Arthritis	
**4**	**Geriatric Syndrome**	12	Fall incidents	Incident Report
		13	Ulcers	Medical History, Progress Notes
		14	Polypharmacy	Medication History
		15	Dysphagia	Medical History, Swallowing Assessment, Meals, Drinks, Nutrition Assessment
		16	Pain	Progress Notes
		17	Fracture	Progress Notes
**5**	**Cognition**	18	Cognition	Medical History
		19	Dementia	Medical History, Progress Notes
**6**	**Nutrition**	20	Weight loss	Progress Note, Weight Chart
**7**	**Activities of Daily Life (ADL)**	21	Dressing, Personal Hygiene, and Toileting	ADL chart
		22	Mobility	Mobility Assessment
**8**	**Elimination**	23	Incontinence (Faecal)	Bowel chart
**9**	**Emotional**	24	Depression	Medical History, Progress Notes
		25	Anxiety	Behaviour assessment
		26	Insomnia	
**10**	**Communication**	27	Vision	Medical History, Progress Notes
		28	Hearing	
**11**	**Other symptoms**	29	Dyspnea	
		30	Anaemia and haematinic deficiency	Progress Notes
		31	Dizziness	
		32	Feet or Foot problems	

Along with identifying the frailty deficits for reFI, data sources within the RACs were also recognised for their computation. Different assessments and observation charts along with the daily progress notes were used to identify the frailty deficits, where the details on the specific data sources can be found in Additional file 1. Unlike clinically coded data (e.g., ICD-9 or ICD-10 diagnostic codes), the data from RACs are primarily composed of free-text notes and a coding guide has been developed to identify the frailty deficits (Additional file 1). The coding guide consisted of a list of keywords (including diseases) that were identified from the medical history recorded in the dataset, along with potential variations and abbreviations identified using the medical vocabulary whose evidence was found in the progress notes. It should be noted that the evidence of reFI deficits reported in this paper was constructed using the data recorded by RFBI, but the proposed reFI could be applied to other RACs as they all record similar data (section Data Description) for adhering to the AACQSC quality standards. The coding guide (Additional file 1) could be extended to add any specific information that any RAC would want to extract based on their needs and requirements. A comprehensive coding guide (if required) could also be easily created using the information available through medical coding standards (e.g., ICD and SNOMED-CT). Common diagnoses among elderly are also available in the literature [35, 36].As the reFI was designed to track the health condition of residents, the temporal characteristics of the frailty deficits were also identified. Deficits that could be addressed by intervention, in order to reverse the health deterioration e.g., pain, polypharmacy, weight loss, etc. were associated with a 6 months history [33, 34, 37–40]. A five-year medical history was considered for the deficits that can permanently impact the health of the residents, e.g., cardiac disease, cancer, thyroid, etc. The details of deficit identification methods are listed in Additional file 1. It should be noted that the deficit identification method was computed in collaboration with the experts in the field, considering the data collection and recording protocol at RFBI RACs. However, the computation method is designed to support scalability to other RACs.

Due to the subjective nature of progress notes and lack of level of health deterioration information in observation charts and assessments, the frailty syndromes were identified as binary variables, i.e., 0 and 1 for absence and presence of frailty deficit respectively. The extent of deterioration (mild or severe) was not covered in quantifying the frailty deficit.

### Participants

EHRs of residents admitted to RFBI from 1 January 2010 to 1 January 2021, aged 65 or over and with a stay period of over 6 months at RACs were included in the study. This limit was enforced as at least 6 months history was required for computing reFI [39, 40]; the remainder of the duration was used to track changes in the frailty index for the residents. It should be noted that the 6 month timeline was selected as it was the shortest time window found in the literature for calculating frailty [33, 34, 37–40]. Moreover, residents with missing data sources, e.g., assessments and observational charts, etc. for more than four non-chronic and -acute diseases frailty deficits (> 15% of the frailty deficits – Additional file 1) were not considered in the study. The inclusion criteria were verified by experts in the field.

The anonymised data consisted of 7181 residents, but only 2588 residents were included in the study that satisfied the criteria mentioned above. There were ≈60% of the residents who had < 1-year stay in RACs, where the average length of stay was 1.2 ± 2.2 years.

### Statistical analysis

Descriptive statistics (mean, standard deviation (SD) and percentage) for reFI were calculated. A kernel density plot was included to visualise the distribution of the reFI. In addition, the reFI was plotted against age, stratified by gender. Frailty status for the residents was designated in four categories (fit, mildly frail, moderately frail, and severely frail) defined by reFI quartiles using the 99th percentile as the upper limit, following the work of Clegg et al. [14] who proposed reFI in the primary care setting.

Survival analysis for 1, 3, and 5 years was performed using the Kaplan-Meier estimator for the four frailty categories, which were compared using the log-rank test. The baseline was established after 6 months from the date of admittance to the RAC (minimum time duration required for computing reFI). Hazard ratios (HRs) were estimated for the outcome of mortality. The Cox regression model was adjusted for age and gender, where reFI (proportion of accumulated deficits to the total number of frailty deficits) was used as the independent variable. The assumption of the proportional hazard was checked using two goodness-of-fit tests (Schoenfeld residuals and log-likelihood tests). Logistic regression was used to assess the association of reFI with survival, adjusted for age and gender. For this purpose, Receiver Operating Characteristics (ROC) curves were used to compute the area under the ROC curve (AUC), where pseudo-R^2^ estimate was used to assess the variability in the mortality explained by reFI. The before-mentioned analyses are commonly used for validating frailty tools [14–18]. We used Python (packages: scikit-learn [41], lifelines [42], and statsmodels [43]) for the analyses.

## Results

**Fig. 1:**
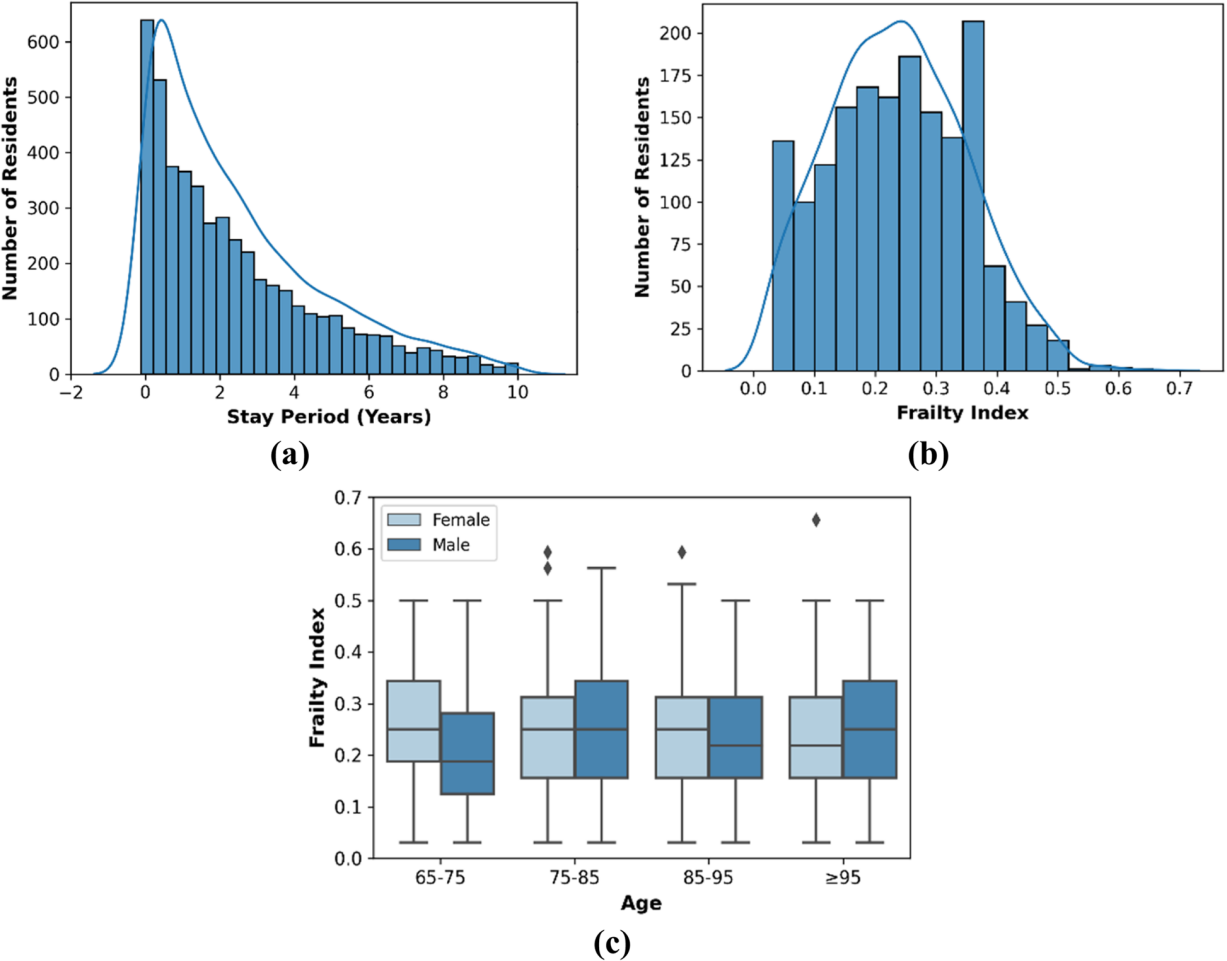
**a** Distribution of resident’s stay period at RFBI RAC, Cohort at baseline (*n* = 2588), (**b**) Distribution of frailty index (reFI) at baseline, (**c**) reFI for different age groups and gender

The primary aim of the proposed reFI was to monitor the health status of the residents, but this required validation of the reFI. As previously mentioned, we performed survival analysis at 1, 3 and 5 years from baseline (6 months from the date of admission) for validation purposes. The distribution of residents’ length of stay at RFBI and the baseline reFI distribution of the cohort are reported in Fig. 1. Details on the baseline characteristics of the cohort are presented in Table 3. Table 3(c) presents sample evidence of frailty deficits extracted from the daily progress notes. The characteristics of the cohort at 1, 3, and 5 years from baseline are reported in Additional file 2 (Table A1 and Fig. A1-A3). The mean reFI at baseline was 0.21 (SD 0.11) and the 99th percentile was 0.5. Therefore, the residents with reFI≤0.13 were defined as fit, 0.13 < reFI≤0.26 as mildly frail, 0.26 < reFI≤0.39 as moderately frail, and reFI> 0.39 as severely frail.

**Table 3:** Baseline characteristics. All values are represented as mean (standard deviation-SD) unless otherwise stated. **(a)** General characteristics **(b)** Frailty domain specific characteristics **(c)** Sample evidence of frailty deficits from daily progress notes

(a)		
**Characteristics**		**Total (n = 2588)**
Age (years)		86.78 (10.15)
Gender	Male	51.82%
	Female	48.18%
Frailty score (reFI)		0.21 (0.11)
reFI - Male		0.21 (0.11)
reFI - Female		0.22 (0.11)
**Frailty Categories**		
Fit		20.17%
Mild Frail		39.57%
Moderate Frail		30.1%
Severe Frail		10.16%
**(b)**		
**Frailty Domains**		**Total (n = 2588)**
Chronic and Acute Diseases		65.65%
Blood-specific Diseases		42.74%
Bone-specific Diseases		54.1%
Geriatric Syndrome		81.3%
Cognition		47%
Nutrition		4%
Activities of Daily Life		52.86%
Elimination		18.59%
Emotional		22.6%
Communication		48.26%
Other symptoms		22.64%
**(c)**		
**Frailty Domains**		**Evidence**
Chronic and Acute Diseases		Seen by Dr. *Deidentified* yesterday. Diagnosis or Bronchitis and COPD. Ordered Antibiotics, Predisolone daily for 4 days, Spiriva, Ventolin and Seretide.
Blood-specific Diseases		Lmo directive for diabetes management signed by Lmo filed in technical nursing.
Bone-specific Diseases		Resident suffers from osteoporosis which causes her chronic back pain. Staff administer analgesia which is prescribed by the lmo.
Geriatric Syndrome		Gp report received, medication changes, Resident is commenced on norspan patch for pain. Pain chart to be commenced to see the effectiveness of pain management
Cognition		Resident’s cognition very poor. Having difficulty communicating difficult to understand what she is saying much of the time. Staff check i/c aid during each care episode.
Nutrition		Resident’s weight attended and documented today due to refusing to eat and concerned of weight loss supervisor inform of weight loss
Activities of Daily Life		Resident’s mobility assisted with wheelchair. Staff required to assist Resident with all aspects of personal hygiene
Elimination		Resident bowel medication withheld tonight due to faecal incontinence.
Emotional		Phone call from resident daughter. Advised daughter that resident has commenced on her antidepressant, aspirin and long term antibiotics for her chronic Urinary Tract Infection.
Communication		Resident is hearing impaired and has trouble communicating, therefore clear, short questions are offered so she can answer with a yes or no.
Other symptoms		Found resident on the floor. Resident stated that while she was walking from her room to dining room she felt dizzy so she just sit on the floor.

**Fig. 2:**
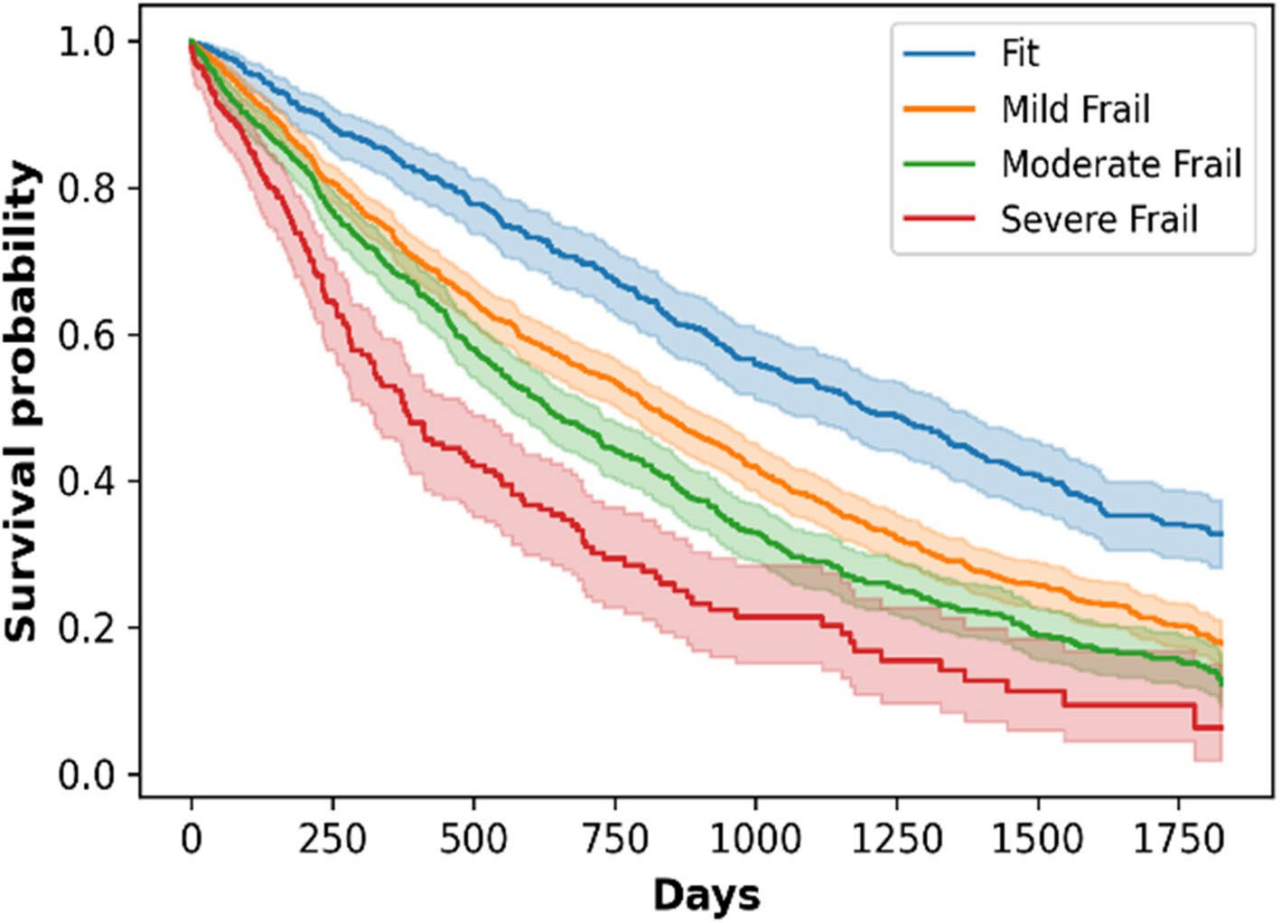
Five-year Kaplan-Meier survival curve for the outcome of mortality for different frailty categories

The survival curves were plotted using the Kaplan-Meier model for each frailty category on the cohort (Fig. 2) and sub-cohort level (based on gender-reported in Additional file 3 -Fig. A1), which shows that the comparatively “*fit*” residents are at lower risk of death than “*severely*” frail residents. It is worth noting here that this cohort is overall frail, considering the case where the adverse event of admission to RAC event has already taken place.

Age and gender-adjusted Cox proportional hazards regression model revealed an association between reFI and mortality (*p* < 0.05). The proportional hazard assumption was supported by the Schoenfeld residuals. Due to the smaller data size as compared to other frailty studies [14, 15, 24, 44], the findings of the Cox regression for frailty sub-categories were not statistically significant. Hence, the analysis was performed at the cohort level. The estimated HRs were 1.12 (95% CI 1.09–1.15), 1.11 (95% CI 1.07–1.14), and 1.1 (95% CI 1.04–1.17) at 1, 3 and 5 years from baseline (Additional file 3, Tables A1-A2). These results demonstrated that for each additional frailty deficit, i.e., 1/32 ≈ 0.03 increase in reFI, the risk of death increased by ≥10% (Additional file 3, Table A2). The unadjusted hazard ratios can also be found in Additional file 3, Table A3.

The ROC analysis of the reFI for mortality outcome showed an AUC of ≥0.60 (with associated lower limit of the 95% confidence interval) for 1, 3 and 5-year mortality (Table 3), where an AUC > 0.5 represents a valid association between covariates and outcome. Psuedo-R^2^ estimates of calibration were low for all cases (Table 4), which was consistent with the findings from [14].

**Table 4:** AUC, associated confidence interval, and pseudo-R^2^ estimates for mortality outcome

Outcome of Mortality	AUC (95% CI)	R^2^
1 Year	0.62 (0.57–0.68)	0.08
3 Year	0.62 (0.54–0.66)	0.09
5 Year	0.63 (0.53–0.73)	0.05

We also repeated the analysis for the traditional frailty categories [32] (≤ 0.1, > 0.1 to ≤0.21, > 0.21 to 0.45, and ≥ 0.45 as fit, mildly frail, moderately frail, severely frail respectively), where we found that the overall outcomes were consistent with the results reported here i.e., comparatively “*fit*” residents are at lower risk of death than “*severely*” frail residents (Additional file – Fig. A2) and the results of the cox-regression were not statistically significant for the traditional frailty categories.

## Discussion and limitations

It is important to identify frailty in elderly people so that personalised care plans are developed to reduce or manage the health deterioration risks, providing them the opportunities to retain their quality of life. Many investigators have developed frailty indicators or indices using clinical, health insurance or CGA-based data [1–6, 45]. To identify people at risk in RACs, we developed a reFI using the daily recorded EHRs at RACs in this study. The dataset comprised routinely collected information of the residents in the form of progress notes, observational charts and assessments. The reFI was developed and validated using the well-established frailty framework [1, 4, 14, 31, 32] and feedback from experts in the geriatric field. The reFI contains 32 frailty deficits that corresponded to 11 frailty domains (Table 2). The reFI was computed and validated using the EHR data from RFBI which consisted of 2588 residents. The average reFI of the dataset was 0.21 which shows that this cohort is overall frail (mild frail 0.13 < reFI≤0.26), whereas other studies have also reported that RAC (nursing) home residents are known to be frail [8]. It should be noted that a 6-month history was selected based on the literature [33, 34, 37–40], but the history timeline could be reduced as per the needs and requirements of RACs for tracking purposes. This should be done with caution as a few frailty deficits are evaluated for their persistence over time such as polypharmacy (require a history of more than 2 weeks to avoid erroneous inclusion of antibiotics in polypharmacy), chronic pain (requires pain issues for consecutive 3 months [46, 47]) and weight loss (weight loss within 6 months [33, 48]).

For validation of reFI, its relationship with risk of mortality was analysed at 1,3 and 5 years from a baseline. AUC ≥0.60 (Table 4) was observed for the aforementioned cases demonstrating an association between reFI and mortality. Many studies [49, 50] have demonstrated higher AUC ≥ 0.70, but these involved use of admission to RAC as one of the adverse events under analysis, which had already taken place in this study. Moreover, we also demonstrated that the degree of frailty significantly influenced the 5-year survival trajectory (Fig. 2).

**Fig. 3:**
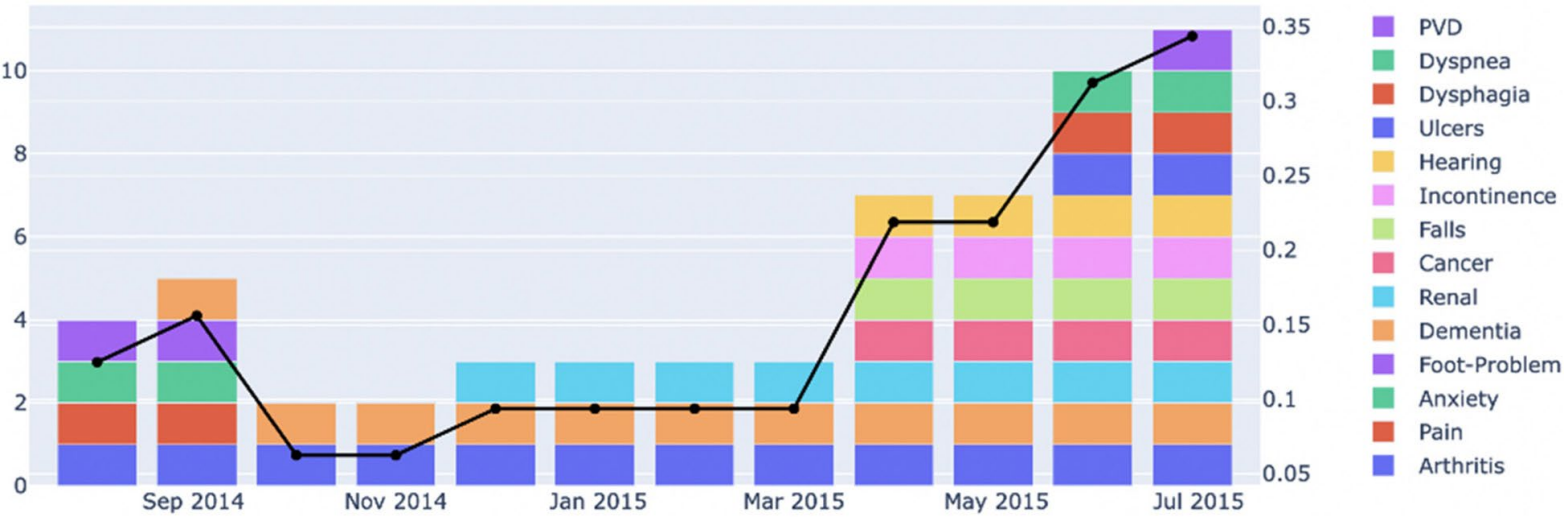
reFI status of a randomly selected resident over 1 year, from August 2014 to July 2015. The left y-axis represents the number of frailty deficits present in the resident, whereas the right y-axis represents the reFI

After validation of the reFI, we used it to monitor the health status of the residents (Fig. 3). This tracking of the changes in reFI not only helps in identifying the residents whose health has deteriorated but also provides information on the changes in frailty indicators that could be used for creating or modifying the personalised care plans for the residents.

A major strength of the study is that the reFI is computed using routinely collected data at RACs. The reFI was developed and validated using the published standard rules of a cumulative FI model, where the frailty deficits were identified from the EHRs with the help of experts in geriatric medicine and care workers at RACs (including GPs, caretakers and nurses). RACs in Australia record this data to comply with the quality standards proposed by AACQSC. Hence, the proposed data-driven reFI could be adopted by any RACs to automate the process of quantifying frailty that complies with the government quality standards. It should also be noted that CGA may be performed if any changes in health conditions are identified using reFI to intervene and manage the health risk. Therefore, reFI could be potentially used as a tool to identify the time for CGA of the residents. This paper demonstrates the utility of reFI for RFBI, but could be applied to other RACs within Australia. Moreover, the proposed reFI has the potential for further international adoption, e.g., RACs in the UK also record the daily routine operational data, which can be used for computing reFI. It should be noted here that the data from one of the Australian RACs were used to demonstrate the validation and utility of reFI. The frailty categories and overall results (e.g. mean reFI) may vary for different countries as the admission criteria to RACs depends on the rules and regulations followed by a country^7^.

To our knowledge, reFI is the first frailty index that tracks the health deficits of residents at RACs. The proposed reFI can be easily and frequently recomputed per resident using real-time routinely operational collected data, eliminating the effort and time consumption in performing CGA. Any changes in the reFI can help in the timely identification of health risks. Research studies have demonstrated that intervention can reverse or delay the progression of frailty [25, 26, 51, 52]. Hence, the changes in the reFI can aid in introducing timely intervention to address, manage or mitigate the frailty risk. reFI can thus serve as an automated tool for computing frailty from the recorded data and identifying changes in the frailty without human intervention. Moreover, aggregated data over all residents in RAC might provide an overview of the care requirements of the residents.

There are important limitations of this study. The FI based on clinical data is typically validated against three adverse outcomes: 1) hospitalisations, 2) admission to RAC and 3) mortality. As the participants in this study were already residents at RAC, this suggests that one of the adverse outcomes has already taken place. The reFI was validated against mortality outcome only as the data for hospitalisations was not available. But it should be noted that the validity of reFI for mortality was comparable with CGA-based FIs [2, 53]. The reFI was also not validated against other clinical eFIs for the same cohort. This was due to the unavailability of clinical and frailty-based CGA data, which requires additional studies to acquire this data. Moreover, the frailty categories were defined based on population quartiles [14]. Hence, the different frailty categories cannot be directly compared to other studies. It should be noted that no change in overall validation results were observed for traditional frailty categories (section Results).

We proposed a reFI for RACs using binary frailty deficits, i.e., either the frailty deficit was present or absent in residents. The frailty deficit cannot identify the severity of the condition (like a weighted frailty deficit), e.g., hearing impairment could be mild requiring a hearing aid in contrast to being unable to hear, but currently the proposed reFI only identifies that a resident has a hearing impairment. Future work aims to address this shortcoming, where the frequency of the data records can be effectively used to identify the severity of the condition. It is worth noting that any FI (weighted or binary) can serve the goal of identifying people at health risk [54]. Moreover, longitudinal analysis would be employed in the future to model and better interpret how the changes in frailty indices over time impact health status and survival.

## Conclusion

We have developed and validated a reFI for RACs using the daily routine operational data recorded for residents. The data is generally recorded to comply with the quality standards; hence the reFI is not dependent on performing specialised assessments. The reFI could facilitate early detection of changes in health conditions that could aid in identifying and addressing the underlying frailty deficit. The utility of the reFI was demonstrated using the data acquired from RFBI (26 RACs), but it has the potential to be adopted on national and international standards.

## Supplementary Information

**Additional file 1.** Description of frailty syndromes and their computation method to estimate reFI using operational data of RACs.

**Additional file 2.** Characteristics and reFI distribution of cohort from RFBI RAC.

**Additional file 3.** Outcome of the Kaplan-Meier survival analysis and cox regression model reveal an association between reFI and mortality.

## Data Availability

The datasets used and/or analysed during the current study cannot be made publicly available as the data was acquired from aged care homes that did not provide consent for their data to be shared publicly but are available from the corresponding author on reasonable request.

## Abbreviations

AACQSC: Australian Aged Care Quality and Safety Commission
ACFI: Aged Care Funding Instrument
AUC: Area Under Curve
CGA: Comprehensive Geriatric Assessments
EHRs: Electronic Health Records
FI: Frailty Index
HR: Hazard Ratio
RACs: Residential Aged Care Homes
reFI: Retrospective electronic Frailty Index
RFBI: Royal Freemasons Benevolent Institution
ROC: Receiver Operating Characteristic
SD: Standard Deviation

## Footnotes

1 https://www.agedcarequality.gov.au/providers/standards/guidance-resources

2 https://www.agedcarequality.gov.au/providers/standards

3 https://www.health.gov.au/news/new-residential-aged-care-quality-indicators

4 https://www.agedcarequality.gov.au/sites/default/files/media/acqsc_aged_care_quality_standards_fact_sheet_4pp_v8.pdf

5 https://www.agedcarequality.gov.au/reports

6 https://www.health.gov.au/initiatives-and-programs/an-acc

7 https://www.health.gov.au/health-topics/aged-care/about-aged-care/what-is-aged-care; https://www.nhs.uk/conditions/social-care-and-support-guide/care-services-equipment-and-care-homes/care-homes/; https://www.medicare.gov/sites/default/files/2019-10/02174-nursing-home-other-long-term-services.pdf
