# Supplementary material for: Development and validation of retrospective electronic frailty index using operational data of aged care homes
Source: BMC Geriatr. 2022 Dec 1;22:922. doi: 10.1186/s12877-022-03616-0 (PMC9714152; doi:10.1186/s12877-022-03616-0)
Supplement: Supplementary file 1 — Additional file 1. Description of frailty syndromes and their computation method to estimate reFI using operational data of RACs. [file 12877_2022_3616_MOESM1_ESM.docx]

**Additional file 1:** Frailty syndromes and their computation method to estimate reFI for RFBI RACs. The number of occurrences can be changed to address different data recording protocols employed in different RACs. Similarly, data source could be different and hence can be changed for different RACs.

| **Ind.** | **Domain** | **Frailty Deficit No. - Name** | | **Keywords for Identification of Frailty Deficit** | **Data Source** | **Timeline to Search** | **Identification Method** |
| --- | --- | --- | --- | --- | --- | --- | --- |
| **1** | **Chronic and Acute Diseases** | 1 - Respiratory | | Chronic Obstructive Pulmonary Disease, COPD, Chronic Obstructive Airways Disease, COAD, Emphysema, Chronic bronchitis, bronchiectasis, Chronic asthma | Medical History OR  Progress Notes | Full history | Single occurrence |
|  |  | 2 - Cardiac | | AF, arrhythmia, atrial, fibrillation, Afib, heart failure, congestive heart failure, CHF, CCF, congestive cardiac failure, congestive heart disease, CHD, heart valve disease, stenosis, valvular insufficiency, tricuspid regurgitation, pulmonary regurgitation, mitral regurgitation, aortic regurgitation, ischaemic heart disease, IHD, ischemic, coronary artery disease, coronary heart disease, CAD, CHD |  |  |  |
|  |  | 3 - Neurological | | Cerebrovascular disease, CVA, Stroke, TIAs, TIA’s, embolism, cerebral infarction, subarachnoid haemorrhage, intracerebral haemorrhage, intracranial haemorrhage, Parkinson, tremor, Parkinsonism, Parkinson’s Disease, stroke, Amyotrophic lateral sclerosis, ALS. |  |  |  |
|  |  | 4 - Renal | | Chronic kidney disease, CVD, kidney, poor renal function, RnFN, renal |  |  |  |
|  |  | 5 - Cancer | | Cancer, tumor |  |  |  |
|  |  | 6 - Peripheral vascular disease | | Peripheral vascular disease, peripheral artery disease, peripheral arterial disease, PAD, PVD |  |  |  |
|  |  | 7- Thyroid | | Thyroid, Hypothyroidism, Hyperthyroidism, Goiter, Thyroiditis |  |  |  |
| **2** | **Blood-specific Diseases** | 8 - Diabetes | | Diabetes, NIDDM, T2DM, DM, IDDM, Diabetes Mellitus, DMII, T2 DM, insulin dependent | Medical History  OR  Progress Notes | Full history | Single occurrence |
|  |  | 9 - Blood-pressure (Hypertension/ Hypotension) | | Hypertension, high blood pressure, HTN, hypotension, low blood pressure, syncope, HoTN. |  |  | Single occurrence in Medical History  OR  At least 3 occurrences in Progress Notes |
| **3** | **Bone-specific Diseases** | 10 - Osteoporosis | | Osteoporosis |  |  | Single occurrence |
|  |  | 11 - Arthritis | | Arthritis, OA, arthrosis, Osteoarthritis, Rheumatoid Arthritis. |  |  |  |
| **4** | **Geriatric Syndrome** | 12 - Falls | | Fall incident report | Incident Report | 6-months | At least 2 occurrences |
|  |  | 13 - Ulcers | | Peptic ulcer, gastric ulcer, stomach ulcer, duodenal ulcer, diabetic ulcer, venous ulcer, pressure ulcer, vascular ulcer | Medical History  OR  Progress Notes | Full history | Single occurrence in Medical History  OR  At least 2 occurrences in Progress Notes |
|  |  | 14 - Polypharmacy | | Daily medication | Medication History | 6-months | Number of medications ≥ 5 for at least 90 days |
|  |  | 15 - Dysphagia | | Dysphagia, difficulty swallowing, swallowing difficulties, difficulty in swallow, swallowing problem, difficult to swallow, poor swallowing, trouble swallowing, swallowing impairment, swallowing difficulty | Medical History, Swallowing Assessment OR  Meals, Drinks and Nutrition Assessment | Full medical history  OR  6-months assessment history | Single occurrence |
|  |  | 16 - Pain | | Pain | Progress Notes | 6-months | At least 2 occurrences for 3 consecutive months^[1, 2]^ |
|  |  | 17 - Fracture | | Fracture | Progress Notes | 6-months | Single occurrence |
| **5** | **Cognition** | 18 - Cognition | | Cognitive impairment, cognitive decline, impaired cogniti*, cognitive deficit', cognitive change, poor cogniti*, decline in cogniti, cognitive dysfunction, cognitive defecit, cognitive deficit, cognitive difficulty, decrease in cogniti* | Medical History | Full history | Single occurrence in medical history |
|  |  | 19 - Dementia | | Dementia | Medical History OR  Progress Notes |  | Single occurrence in medical history  OR  At least 2 occurrences in the progress notes |
| **6** | **Nutrition** | 20 - Weight loss | | Anorex*, weight loss, cachexia, appetite loss, poor appetite. | Progress Notes+ Weight Chart | 6-months | Single occurrence for 2 months |
| **7** | **Activities of Daily Life** | 21 - | Dressing | Assistance required | ADL chart | 6-months | Majority vote (2 or more) |
|  |  |  | Personal hygiene |  |  |  |  |
|  |  |  | Toileting |  |  |  |  |
|  |  | 22 - Mobility | | Assistance required for:  1) Chair-to-chair transfer  2) Sit-to-stand transfer  3) Bed-mobility  4) Wheelchair | Mobility Assessment |  |  |
| **8** | **Elimination** | 23 - Incontinence (Faecal) | | Incontinent | Bowel chart |  | Incontinent for a majority of the recorded days |
| **9** | **Emotional** | 24 - Depression | | Depression, depress*, antidepressant, anti depressants | Medical History  OR  Progress Notes | Full medical history  OR  6-months progress notes | Single occurrence in medical history  OR  At least 2 occurrences for 3 consecutive months |
|  |  | 25 - Anxiety | | Anxiety (search term: anxi*), panic, phob. | Behaviour assessment | 6-months | Single occurrence |
|  |  | 26 - Insomnia | | Insomnia, cannot (can’t, cant) sleep, not sleep, restless sleep, sleep apnoea, restless legs, RLS, difficulty (difficulties) in sleep, sleep assessment |  |  |  |
| **10** | **Communication** | 27 - Vision | | Visual impairment, blindness, Glaucoma, Cataracts, poor vision, macular degeneration, vision loss, vision impairment, low vision | Medical History  OR  Progress Notes | Full history | Single occurrence in medical history  OR  At least 3 occurrences in the progress notes |
|  |  | 28 - Hearing | | Hearing assessment, hearing impairment, impaired hearing, hearing aids, hearing loss, deaf |  |  |  |
| **11** | **Other symptoms** | 29 - Dyspnea (shortness of breath) | | Dyspnea, shortness of breath, SOB. |  |  |  |
|  |  | 30 - Anaemia and haematinic deficiency (B12/Iron deficiency) | | Anaemia, haematinic, anemia, B12, B 12, iron deficiency, haematinic, iron tablet. | Progress Notes | 6-months | At least 2 occurrences for 3 months |
|  |  | 31 – Dizziness | | Dizziness, dizzy, giddiness, light head*, heavy head*, vertigo. |  |  |  |
|  |  | 32 - Feet or Foot problems | | Foot, feet, ankle, heel. |  |  |  |
