# Supplementary material for: Development and validation of retrospective electronic frailty index using operational data of aged care homes
Source: BMC Geriatr. 2022 Dec 1;22:922. doi: 10.1186/s12877-022-03616-0 (PMC9714152; doi:10.1186/s12877-022-03616-0)
Supplement: Supplementary file 2 — Additional file 2. Characteristics and reFI distribution of cohort from RFBI RAC. [file 12877_2022_3616_MOESM2_ESM.docx]

**Additional file 2**

**Table A1:** Characteristics of cohort at 1, 3 and 5 years from baseline.

| **Characteristics** | | **Year 1**  **Total (n=1683)** | **Total (n=1165)** | **Total (n=471)** |
| --- | --- | --- | --- | --- |
| Age (years) | | 87.29 (10.21) | 86.27 (11.93) | 86.22 (12.03) |
| Gender | Male | 52.05% | 51.8% | 51.76% |
|  | Female | 47.95% | 48.2% | 48.24% |
| Frailty score (reFI) | | 0.24 (0.11) | 0.26 (0.11) | 0.3 (0.1) |
| reFI - Male | | 0.23 (0.11) | 0.26 (0.11) | 0.3 (0.1) |
| reFI - Female | | 0.24 (0.11) | 0.26 (0.11) | 0.3 (0.1) |
| **Frailty Categories** | | | | |
| Fit | | 14.02% | 8.58% | 3.18% |
| Mild Frail | | 36.13% | 22.32% | 17.41% |
| Moderate Frail | | 35.53% | 65.41% | 73.67% |
| Severe Frail | | 14.32% | 3.69% | 5.73% |
| **Frailty Domains** | | | | |
| Chronic and Acute Diseases | | 72.61% | 77.51% | 84.5% |
| Blood-specific Diseases | | 45.4% | 47.73% | 58.81% |
| Bone-specific Diseases | | 62.57% | 68.5% | 78.34% |
| Geriatric Syndrome | | 86.7% | 91.59% | 93.2% |
| Cognition | | 52.25% | 57% | 64.33% |
| Nutrition | | 2.86% | 3.1% | 1.7% |
| Activities of Daily Life | | 56.44% | 58.62% | 63.27% |
| Elimination | | 22.64% | 27.38% | 32.48% |
| Emotional | | 19.13% | 20.86% | 17.2% |
| Communication | | 56.92% | 61.55% | 74.52% |
| Other symptoms | | 23.12% | 23.1% | 25.7% |

| 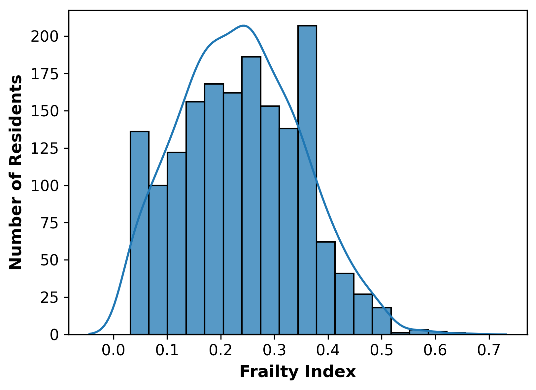  **(a)** | 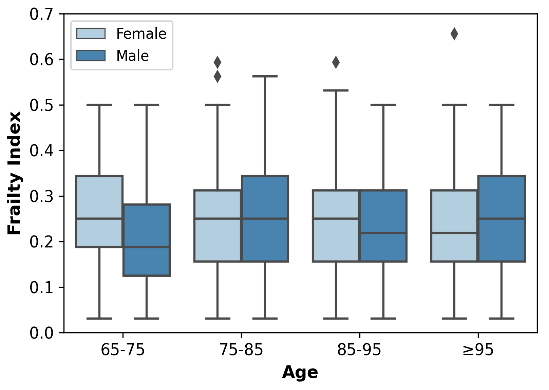  **(b)** |
| --- | --- |

**Figure A1:** Cohort at 1 year from baseline (n=2588), (a) Distribution of frailty index (reFI) at baseline, (b) reFI for different age groups and gender

| 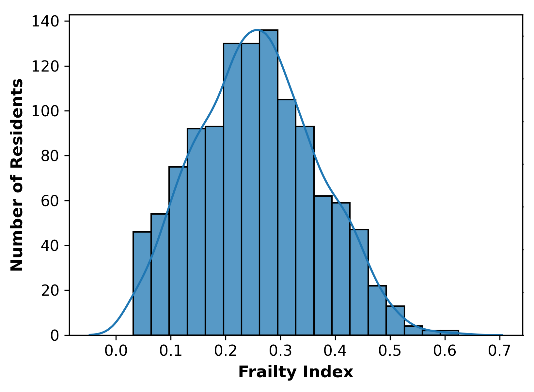  **(a)** | 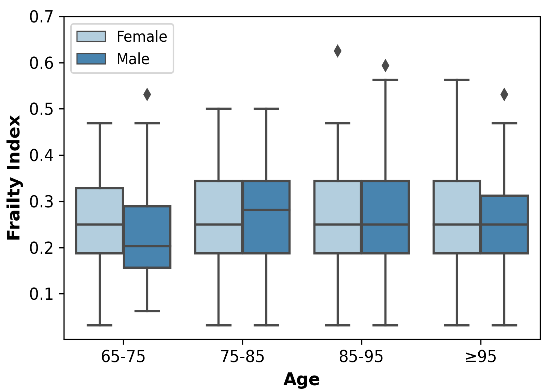  **(b)** |
| --- | --- |

**Figure A2:** Cohort at 3 year from baseline (n=2588), (a) Distribution of frailty index (reFI) at baseline, (b) reFI for different age groups and gender

| 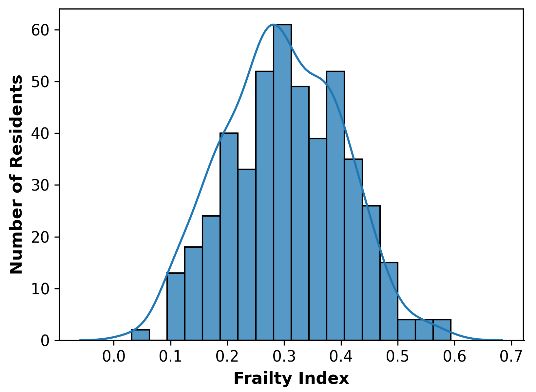  **(a)** | 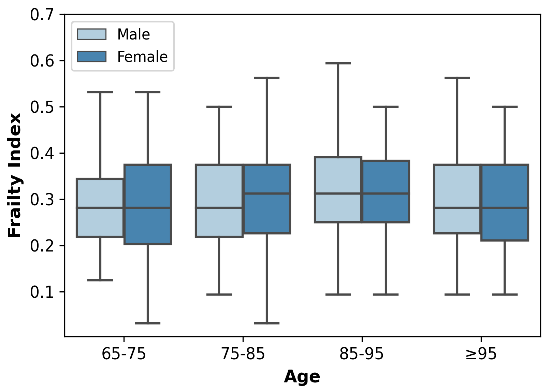  **(b)** |
| --- | --- |

**Figure A3:** Cohort at 5 year from baseline (n=2588), (a) Distribution of frailty index (reFI) at baseline, (b) reFI for different age groups and gender
