# Supplementary material for: Development and validation of retrospective electronic frailty index using operational data of aged care homes
Source: BMC Geriatr. 2022 Dec 1;22:922. doi: 10.1186/s12877-022-03616-0 (PMC9714152; doi:10.1186/s12877-022-03616-0)
Supplement: Supplementary file 3 — Additional file 3. Outcome of the Kaplan-Meier survival analysis and cox regression model reveal an association between reFI and mortality. [file 12877_2022_3616_MOESM3_ESM.docx]

**Additional file 3**

| **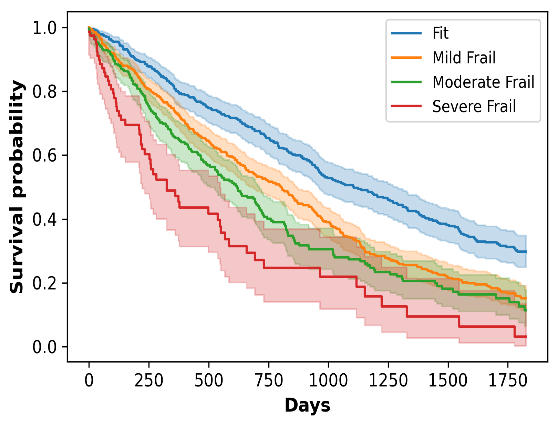**  **(a)** | **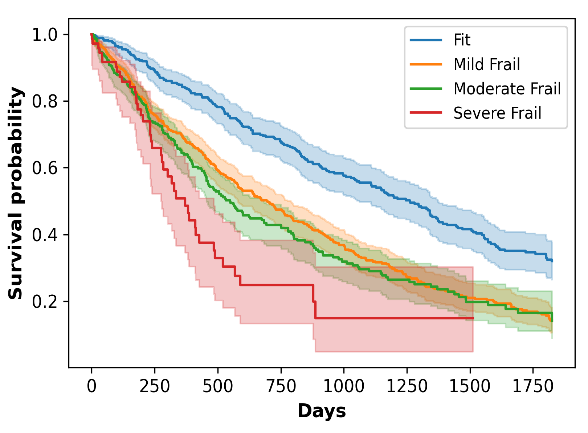**  **(b)** |
| --- | --- |

**Figure A1:** Five-year Kaplan-Meier survival curve for the outcome of mortality for (a) male, and (b) female cohorts

**Table A1:** Age and Gender adjusted 1, 3 and 5 year hazard ratios for one unit increase in the reFI

| **Years after baseline** | **Hazard Ratio** | **Lower Bound** | **Upper Bound** | **P-value** |
| --- | --- | --- | --- | --- |
| 1 | 43.65 | 17.58 | 108.34 | <0.01 |
| 2 | 30 | 10.4 | 86.64 | <0.01 |
| 3 | 25.67 | 3.61 | 182.34 | <0.01 |

**Table A2:** Age and Gender adjusted 1, 3 and 5 year hazard ratios for 0.03 unit increase in the reFI

| **Years after baseline** | **Hazard Ratio** | **Lower Bound** | **Upper Bound** | **P-value** | **Risk of death** |
| --- | --- | --- | --- | --- | --- |
| 1 | 1.12 | 1.11 | 1.1 | <0.01 | 12% |
| 2 | 1.09 | 1.07 | 1.04 | <0.01 | 11% |
| 3 | 1.15 | 1.14 | 1.17 | <0.01 | 10% |

**Table A3:** Unadjusted 1, 3 and 5 year hazard ratios for 0.03 unit increase in the reFI

| **Years after baseline** | **Hazard Ratio** | **Lower Bound** | **Upper Bound** | **P-value** |
| --- | --- | --- | --- | --- |
| 1 | 1.12 | 1.11 | 1.11 | <0.01 |
| 2 | 1.1 | 1.07 | 1.05 | <0.01 |
| 3 | 1.15 | 1.14 | 1.18 | <0.01 |

**
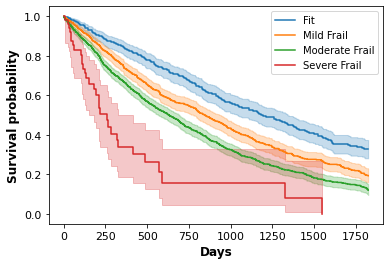
**

**Figure A2:** Five-year Kaplan-Meier survival curve for the outcome of mortality for traditional frailty categories (≤ 0.1, >0.1 to ≤0.21, >0.21 to 0.45, and ≥0.45 as fit, mildly frail, moderately frail, severely frail respectively)
